# Distribution of angle lambda and pupil offset as measured by combined Placido Scheimpflug Topography

**DOI:** 10.1007/s10792-022-02394-3

**Published:** 2022-07-28

**Authors:** Hesham Mohamed Gharieb, Hisham Samy Shalaby, Ihab Saad Othman

**Affiliations:** 1 Department of Ophthalmology, Faculty of Medicine, Ain Shams University, Abbassia Square, Cairo, 11591 Egypt; 2 Eye World Hospital, Giza, Egypt; 3 Faculty of Medicine, Cairo University, Giza, Egypt

**Keywords:** Angle lambda, Angle kappa, Pupil offset, Sirius, Placido Scheimpflug topography

## Abstract

**Background:**

Angle lambda is the angle between the pupillary axis and the line of sight. It is important for accurate centration during anterior segment surgery. The purpose of this study is to identify the distribution of angle lambda and pupil center offset as measured by a combined placido disc Scheimpflug topography system.

**Methods:**

A prospective non-randomized study was performed on 2178 eyes in Eye World Hospital, Giza, Egypt. Sirius device (CSO, Costruzione Strumenti Oftalmici, Florence, Italy, version 3.2.1.60) was used to measure average keratometry (K), anterior chamber depth (ACD), central corneal thickness (CCT), horizontal visible iris diameter (HVID), pupil radius (PR), pupil center intercept x-component (PCI-x), and pupil center intercept y-component (PCI-y). Axial length (AL) was measured by immersion A-scan Eyecube Ultrasonography device (Ellex, Adelaide, South Australia, Australia). Angle lambda was calculated by a trigonometrical equation. Pearson correlation was used to analyze the correlation between angle lambda and age and refraction.

**Results:**

Average angle lambda in all eyes was 3.32° ± 1.99. Mean angle lambda was significantly smallest in myopia and largest in hyperopia. Age correlation to angle lambda was insignificant. Average PCI-x and PCI-y in all eyes was − 0.047 mm and + 0.091 mm, respectively.

**Conclusions:**

Angle *λ* is significantly larger in hyperopia than myopia, and the effect of age is insignificant. Pupil center offset was horizontally greater in hyperopia than in myopia. We therefore encourage the preoperative assessment of angle *λ* to avoid decentered ablation, especially when treating hyperopia.

## Introduction

Angle lambda (*λ*) is the angle between the pupillary axis (line passing through the center of the pupil perpendicular to the center of curvature of the cornea) and the line of sight (which passes from the point of fixation through the center of the pupil), meeting at the center of the pupil [1]. It is often confused with angle kappa (*κ*), which is the angle between the visual axis (line joining the point of fixation to the fovea) and the pupillary axis, meeting at the nodal point [2]. Nonetheless, the difference between them is minute [3], and are considered equivalent when fixating on a distant target [4], and they are often mentioned interchangeably [5]. According to Donders, the size of angle *λ* ranges from 3.5° to 6°, averaging at 5.082° in emmetropes [6].

The clinical significance of angle *λ* is for accurate centration during anterior segment surgery. A large angle may lead to photoablation displacement in refractive procedures [7], graft decentration during keratoplasty [8], and decentration of multifocal intraocular lenses [9]. In addition, angle *λ* is considered to be a diagnostic detector for squint and albinism [10, 11]. Moreover, through device misalignment, angle *λ* could cause inaccurate measurement of corneal aberrations by corneal topography with videokeratoscopy. Many research laboratories use videokeratoscopy in combination with Shack–Hartmann aberrometry to study high order ocular aberrations. But while the Shack–Hartmann aberrometer is aligned coaxially with the line of sight, videokeratoscopes are not. When angle *λ* is more than 2°–3°, the misalignment can lead to incorrect measurements of optical aberrations [12].

Angle *λ* can be identified by clinically by observing the slight nasal displacement of the corneal light reflex [5]. The synoptophore [7], the perimeter [13] arc and more recently video gaze trackers [14] can give numerical readings of the angle. All such techniques depend on measuring the distance between the first Purkinje corneal image (which is actually a virtual image behind the cornea) and the pupillary center [14].

Scheimpflug cameras generate a cross-sectional image of the anterior chamber. Angle *λ* is calculated by measuring the iris plane inclination relative to the target of fixation [7–15]. On the other hand, Placido–ring-based topographers read the distance between the pupillary center and the center of the Placido ring [16].

The Sirius device (CSO Costruzione Strumenti Oftalmici, Florence, Italy) is a combination of a monochromatic 360° rotating Scheimpflug camera and a Placido disk-based corneal topographer. The system can measure 35,632 points from the anterior cornea and 30,000 points from the posterior corneal surface and produces highly reliable anterior segment measurements [17].

The aim of this study is to illustrate the distribution of angle *λ* and pupillary center offset readings as obtained using the Sirius device and to analyze the angle with respect to age and refractive state.

## Materials and methods

A prospective non-randomized study was performed in Eye World Hospital, Giza, Egypt, after approval by Cairo University Ethical Committee and in accordance with the 1975 Helsinki declaration. The study was conducted between May 2020 and March 2021. A written informed consent was taken from each participant (or their legal guardians for minors), before recruitment. The eyes of 1089 participants were recruited from patients visiting the Eye World Hospital. We included subjects at least 8 years of age or older (to ensure cooperativeness), with refraction within range from − 24.00 to + 7.00 diopter subjective sphere and up to -6.00 diopters subjective cylinder. Exclusion criteria were any corneal pathology (including dry eye), previous ocular surgery, and history of contact lenses wear within one month before the study. All subjects underwent full ophthalmic examination including best corrected visual acuity, refraction, and slit-lamp biomicroscopy. Axial length (AL) was measured under topical anesthesia by immersion technique of A-scan Eyecube Ultrasonography device (Ellex, Adelaide, South Australia, Australia). Ten serial readings of axial length were taken. The axial length was automatically measured from the first ultrasound spike representing anterior surface of cornea to the vitreous-retina interface spike.

All readings by the Sirius (CSO, Costruzione Strumenti Oftalmici, Florence, Italy, version 3.2.1.60) were captured between 3 and 7 pm with the participant awake for at least 6 h before acquisition [18]. Subjects were asked to maintain an upright head posture and a straight gaze. Four shots were taken by a single experienced optician using automatic acquisition and the image with the highest quality factor was selected. The following data was recorded for each participant: average keratometry (K), anterior chamber depth (ACD), central corneal thickness (CCT), horizontal visible iris diameter (HVID), pupil radius (PR), pupil center intercept x-component (PCI-x), pupil center intercept y-component (PCI-y).

### Calculating angle lambda

**Fig. 1:**
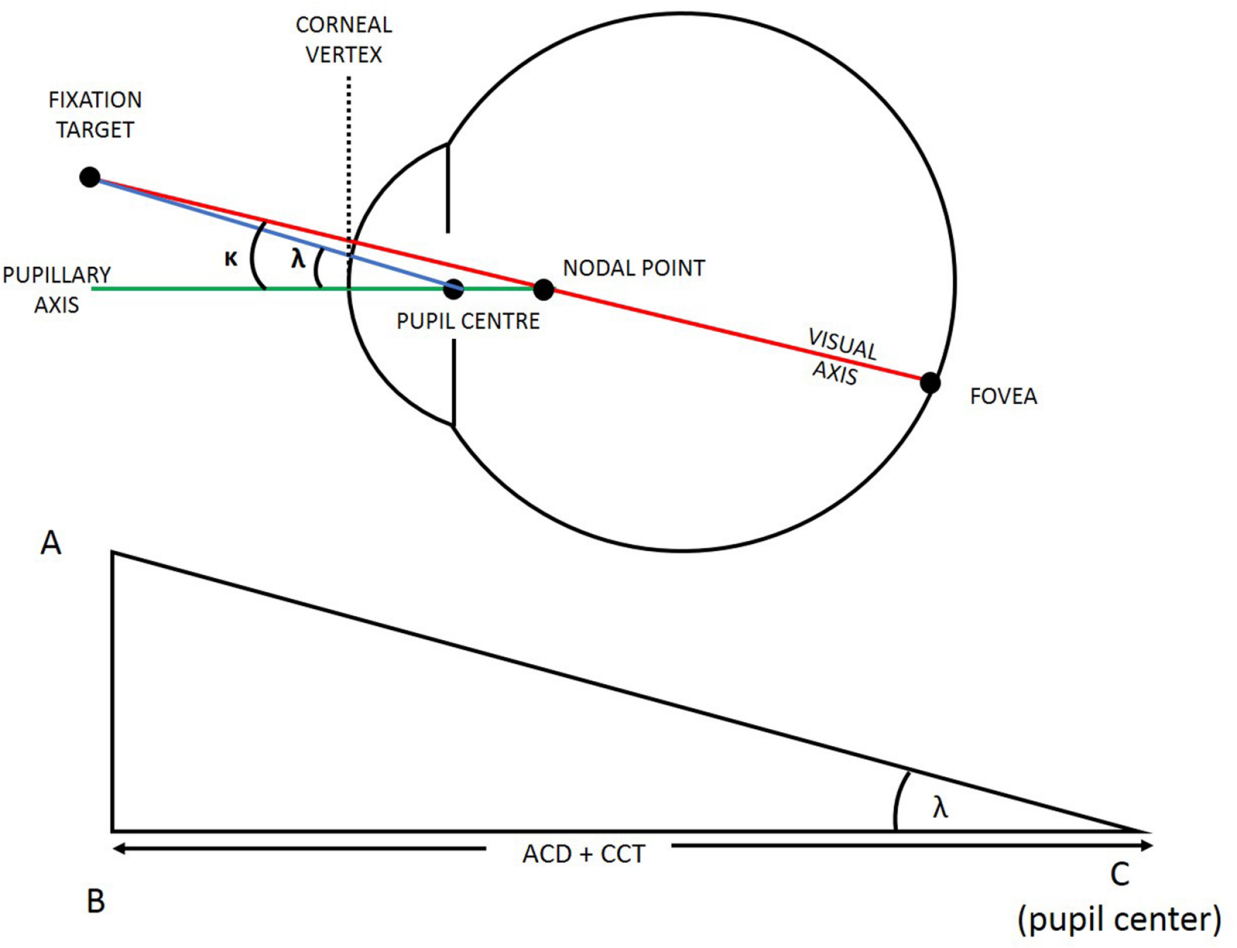
Calculating angle lambda

**Fig. 2:**
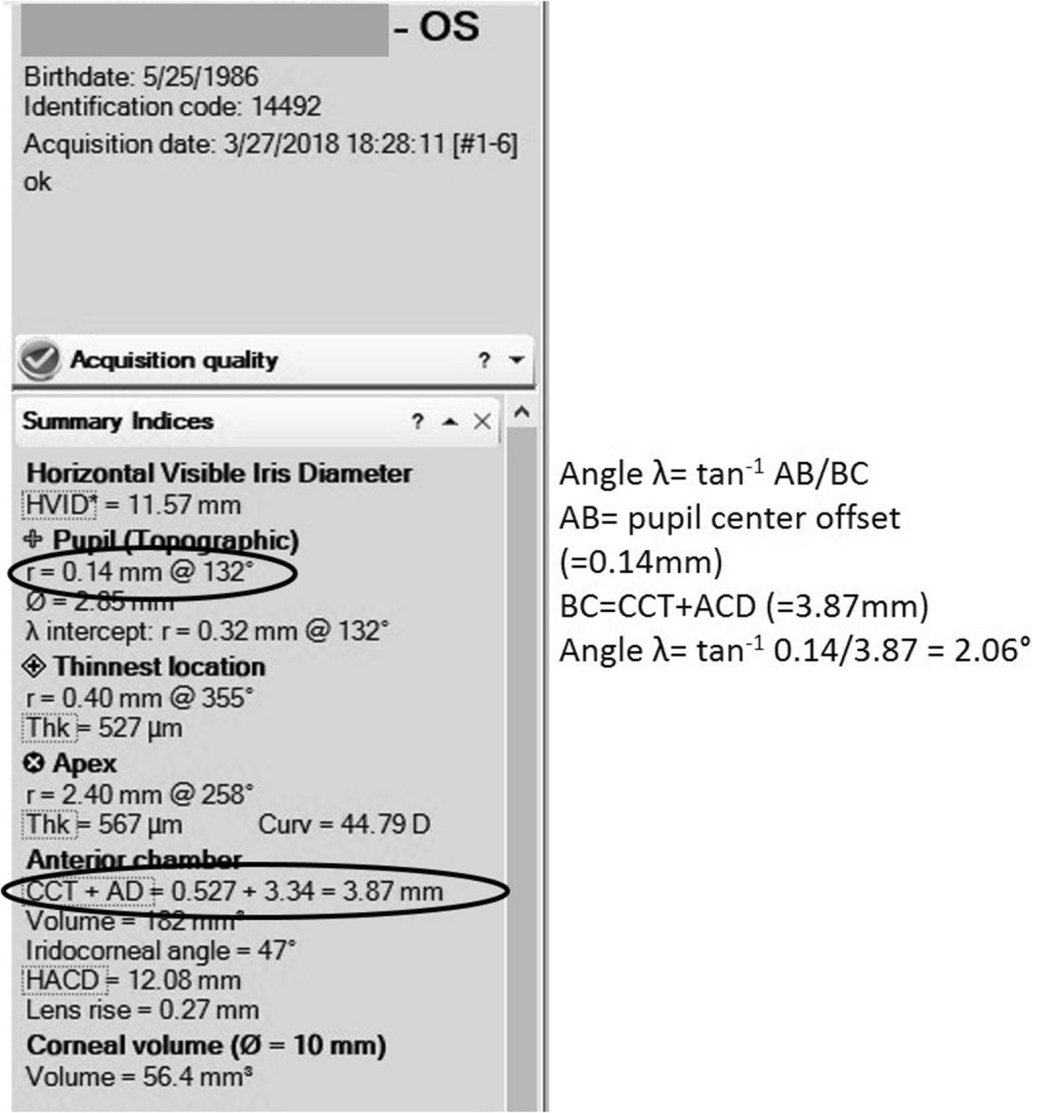
Screenshot of Sirius measurements and calculation of angle lambda

As shown in Fig. 1, ABC is a right-angled triangle made of three points: the point on the anterior corneal surface intersecting with the line of sight (A), the point on the anterior corneal surface intersecting with the pupillary axis (B), and the center of the pupil (C). The angle subtended at the meeting of AC with BC is angle *λ*. Line BC is adjacent to angle *λ* and is calculated by adding the ACD to the CCT (both measured by the Sirius device). Meanwhile line AB is opposite to angle *λ* and represents the pupil center offset (as measured by the Sirius device). Therefore, applying a trigonometrical principle previously described in other studies [3–19], angle *λ* = tan^−1^ AB/BC. A screenshot of the Sirius device measurements and an example of the calculation of angle *λ* are shown in Fig. 2.

### Statistical analysis

Data were statistically analyzed, and graphs were plotted using MedCalc Statistical Software version 18.9.1 (MedCalc Software bvba, Ostend, Belgium). Shapiro–Wilk test was used to test the normality distribution of the data. Age and gender were described as frequency while other data were described as mean ± SD (range) with 95% confidence interval (95% CI). Emmetropia was defined as SE from + 0.50 to − 0.50 diopters, myopia as SE less than − 0.50 diopters and hyperopia as SE more than + 0.50 diopters. Comparison between different refraction groups and different age groups was done using ANOVA with Post hoc test. Pearson correlation coefficient was used to analyze the correlation between various factors and angle *λ*. Univariate regression analysis was used to determine the impact of each independent variable on angle *λ*, and multivariate regression analysis was then done using a stepwise approach to include variables that actively participate in angle *λ* determination and exclude less important variables. Graphs were created using Microsoft Excel 356 program (Redmond, Washington, USA).

## Results

Our study was conducted on 2178 eyes of 1089 subjects. This included 630 males (57.9%) and 459 females (42.1%), and 1097 right eyes (50.4%) and 1081 left eyes (49.6%). Demographic and clinical data for our study population are presented in Table 1.

**Table 1:** Demographic and clinical data for study population (SE: spherical equivalent, BCVA: best corrected visual acuity, K: keratometry, CCT: central corneal thickness, ACD: anterior chamber depth, HVID: horizontal visible iris diameter, PR: pupil radius, PCI-x: pupil center intercept x-component, PCI-y: pupil center intercept y-component, AL: axial length)

	Mean (SD)	Range	95% CI
Age (years)	28.9 (8.7)	8–69	27–28
Subjective Sphere (diopters)	− 2.48 (2.6)	+ 6.5 to − 22.5	− 2.25 to − 2.000
Subjective Cylinder (diopters)	− 1.3 (1)	− 0.25 to − 6	− 1.00 to − 1.000
SE (diopters)	− 3.13 (2.68)	+ 6.25 to − 23.37	− 2.9 to − 2.750
BCVA (decimals)	0.9 (0.3)	0.7 to 1.5	0.9 to 1.000
Average K (mm radius)	7.7 (0.27)	7.00 to 9.09	7.69 to 7.72
CCT (µm)	540 (35)	438 to 643	536 to 540
ACD (mm)	3.2 (0.3)	2.03 to 4.15	3.17 to 3.2
HVID (mm)	12.0 (0.5)	10.48 to 13.1	11.85 to 12.2
PR (mm)	1.9 (0.33)	1.07 to 3.09	1.88 to 1.91
PCI-x (mm)	− 0.05 (0.46)	− 1.94 to 1.4	− 0.1 to − 0.03
PCI-y (mm)	0.09 (0.32)	− 1.47 to 1.38	0.11 to 0.14
AL (mm)	24.8 (1.03)	21.2 to 32.5	24.67 to 24.76
Calculated Angle *λ* (°)	3.32 (1.99)	0.14 to 15.6	2.91 to 3.08

### Distribution of angle lambda

**Fig. 3:**
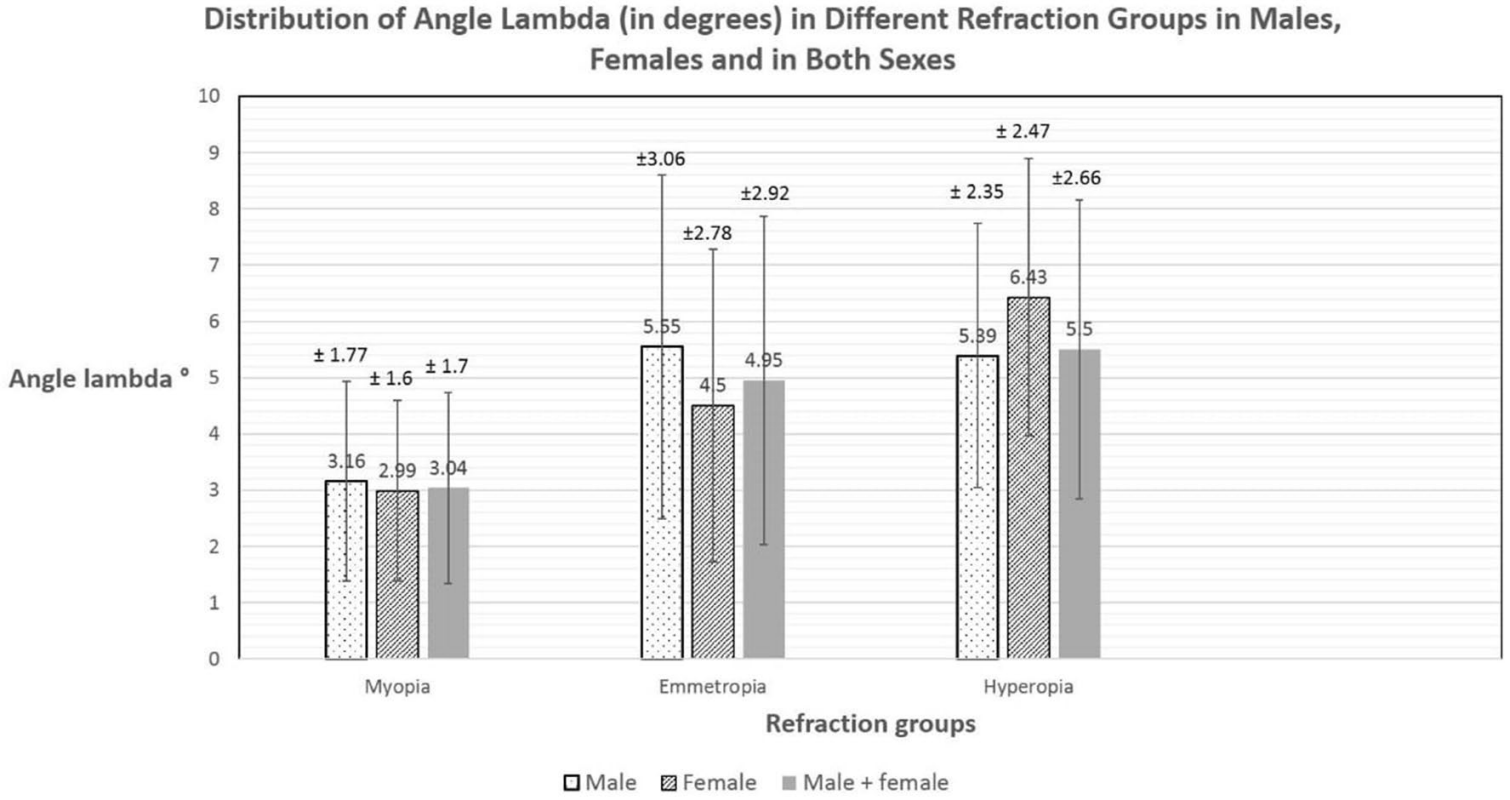
Distribution of angle lambda in degrees in different refraction groups in males, females and in both sexes

**Fig. 4:**
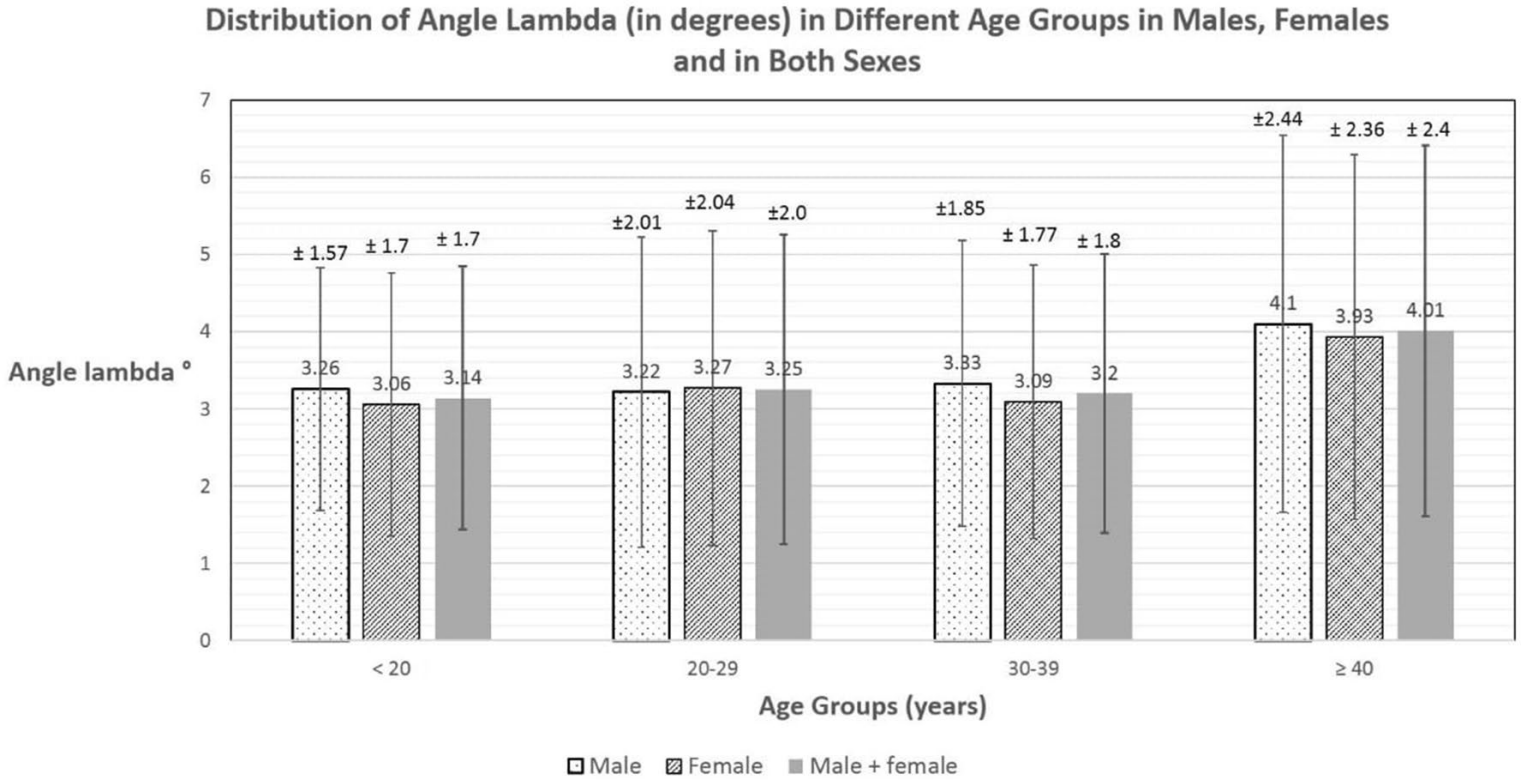
Distribution of angle lambda in degrees in different age groups in males, females and in both sexes

Mean calculated angle *λ* in all eyes was 3.32° ± 1.99° SD. The mean was 3.4° ± 2° SD in males and 3.26° ± 2° SD in females. The distribution of angle *λ* in different groups of the population is seen in Figs. 3 and 4. In different refraction groups (Fig. 3), mean angle *λ* was smallest in myopia (1876 eyes; 3.04° ± 1.7 SD), followed by emmetropia (77 eyes; 4.95° ± 2.92 SD) and largest in hyperopia (206 eyes; 5.5° ± 2.66 SD). The differences between the three refraction groups were statistically significant (*P* < 0.05). In different age groups (Fig. 4), mean angle *λ* was smallest for age group younger than 20 years (393 eyes; 3.14° ± 1.7 SD), 3.25° ± 2.0 SD for age group 20–29 years, 3.2° ± 1.8 SD for age group 30–39 years and largest for age group 40 years and older (4.01° ± 2.4 SD). However, these differences were statistically insignificant (*P* > 0.05).

Pearson correlation results (Table 2) indicate a poor positive correlation between angle *λ* and age, SE, subjective sphere, average K and CCT. In addition, there was a poor negative correlation between angle *λ* and HVID, PR and AL. Exceptionally, there was a medium negative correlation between angle *λ* and ACD (*r* = − 0.4).

**Table 2:** Pearson correlation and regression analysis between angle lambda and independent variables (SE: spherical equivalent, K: keratometry, CCT: central corneal thickness, ACD: anterior chamber depth, HVID: horizontal visible iris diameter, PR: pupil radius, AL: axial length)

	Pearson correlation coefficient		Univariate regression analysis			Multivariate regression analysis		
	*R*	*P* value	Coefficient	*P* value	*R*^2^	Coefficient	*P* value	*R*^2^
Age	0.09	< 0.0001	0.021	< 0.0001	0.008	–	–	0.2125
SE	0.197	< 0.0001	0.146	< 0.0001	0.039	− 0.362	< 0.0001	
Subjective sphere	0.23	< 0.0001	0.17	< 0.0001	0.053	0.588	< 0.0001	
Average K	0.179	< 0.0001	1.317	< 0.0001	0.032	–	–	
CCT	0.023	0.293	0.001	0.293	0.0005	–	–	
ACD	− 0.4	0.0001	− 2.64	< 0.0001	0.16	− 2.456	< 0.0001	
HVID	− 0.029	0.315	− 0.021	0.315	0.0005	–	–	
PR	− 0.012	0.579	− 0.069	0.579	0.0001	0.64	< 0.0001	
AL	− 0.102	0.0001	− 0.2	< 0.0001	0.01	0.381	< 0.0001	

Univariate regression analysis denotes a significant impact (*P* < 0.05) of age, SE, subjective sphere, average *K*, ACD and axial length on angle *λ*. This impact was positive for age, SE, subjective sphere and average *K*, and negative for ACD and AL. Multivariate regression analysis (*R*^2^ of 0.2125) showed that the most important determinants of angle *λ* in this study were SE, subjective sphere, ACD, PR and AL (*P* < 0.0001). This is shown in Table 2.

### Distribution of pupil center offset

**Fig. 5:**
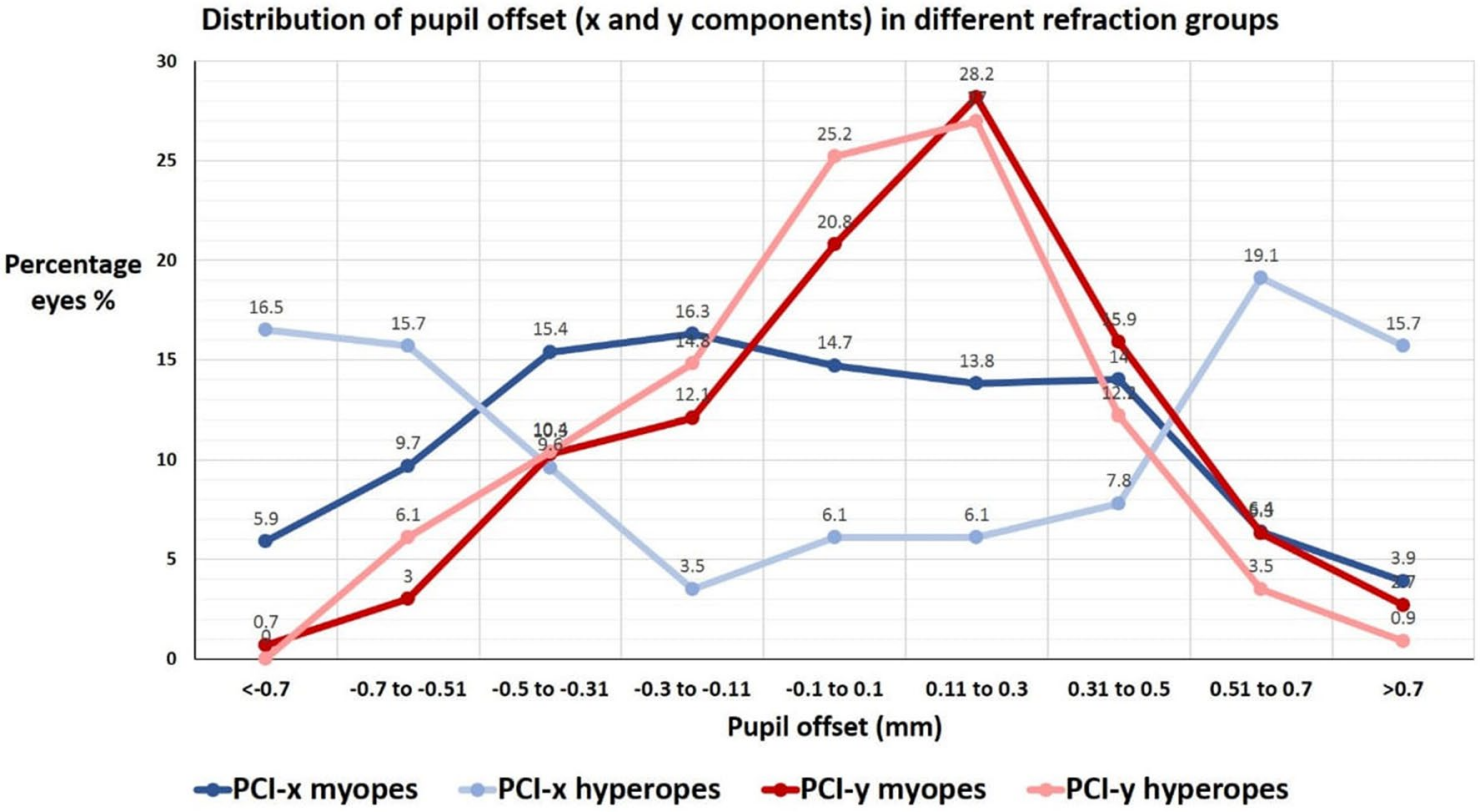
Distribution of pupil offset *X* and *Y*-components in millimeters for myopic and hyperopic eyes (PCI-x: pupil center intercept *x*-component, PCI-y: pupil center intercept *y*-component)

Average PCI-x and PCI-y in all eyes were − 0.047 mm (± 0.46 SD) and + 0.091 mm (± 0.32 SD), respectively (Table 1). The distribution of PCI in different refraction groups of the population is seen in Fig. 5. The distribution for the X-component was symmetrical unimodal in myopia (i.e., bell curve configuration) and symmetrical bimodal in hyperopia (i.e., inverted bell curve configuration). This indicates that pupil center shows more horizontal decentration in hyperopia than in myopia. The distribution of the Y-component was unimodal and slightly skewed to the left in both myopia and hyperopia, denoting that the pupil center shows slightly more upper than lower vertical decentration.

## Discussion

Proper centration is a vital factor for surgical success in refractive surgery, which dictates that ophthalmologists take angle *λ* into consideration preoperatively. This is of utmost value in patients with a large angle *λ* [20]. Uozato and Guyton [8] proclaimed that since photoreceptors are directed toward the center of the pupil, therefore the pupil center should be used for centration. Contrarily, Pande and Hillman [21] described the corneal intercept of the visual axis as the best point of centration because the visual axis joins the fovea to the fixation point. They decided that it was best represented on the cornea by the coaxial corneal light reflex. Nepomuceno et al. [22] reported that centering laser ablation on the pupil center in hyperopes who have a large angle *κ* would result in significant decentration.

In strabismus, accurate angle *λ* measurement avoids under-estimation or over-estimation of the amount of deviation [11]. Even with prescription glasses, a large angle *λ* may induce an undesirable prismatic effect [2].

Average angle *λ* in our study was 3.32° ± 1.99°. This is slightly different from the value reported by London and Wick [3], who calculated angle *λ* in adults to be 5.08°. However, they had not taken measurements of ocular dimensions themselves and had only implemented a mathematical model using corneal curvature measurements and anterior chamber depths recorded by other studies [23, 24]. Similarly, Donders described the size of angle *λ* as ranging from 3.5° to 6°, averaging at 5.082° in emmetropes [6] (Table 3).

**Table 3:** Literature review for studies measuring angle *λ* or *κ*

Study	Angle measured (*λ* or *κ*)	Mean angle value (°)
Current study	Λ	3.32° ± 1.99°
London and Wick (1982) [3]	Λ	5.08°
Donders (1864) [6]	Λ	3.5° to 6° (5.082° in emmetropes)
Yeo et al. (2017) [19]	Κ	3.98° ± 1.12° by Orbascan II 3.19° ± 1.15° by corneal topography and ultrasound biomicroscopy
Hashemi et al. (2010) [2]	Κ	5.46° ± 1.33°
Gharaee et al. (2014) [25]	Κ	4.96° ± 1.38°
Reinstein et al. (2021) [26]	Κ	5.28° ± 1.49° in myopia 5.77° ± 1.29° in emmetropia 6.14° ± 1.44° in hyperopia
Schaeffel (2002) [14]	Κ	3.91° ± 2.73° right eyes 3.93° ± 2.68° left eyes

Angle *λ* and angle *κ* have been described as similar especially when fixating on a distant target^4^. Accordingly, our measurements for angle *λ* are comparable to those of Yeo et al. [19], who measured angle *κ* using two methods: by using an Orbscan II device, and by calculations based on corneal topography and ultrasound biomicroscopy. Angle *κ* averaged 3.98° ± 1.12° with the former and 3.19° ± 1.15° with the latter. Two studies used an Orbscan II device to measure angle *κ* in the Iranian population, and mean angle *κ* was recorded at 5.46° ± 1.33° by Hashemi et al. [2] and 4.96° ± 1.38° by Gharaee et al. [25] The relatively smaller mean angle *λ* calculated in our study could be explained by the fact that over 90% of our population sample were myopes, and angle *λ* is well known to be smaller in myopia and larger in hyperopia [2]. Moreover, the smaller mean angle *λ* calculated in our study could be attributed to the difference in mean age between our population sample (28.9 years) compared to other studies (36.4 years in the study by Yeo et al. and 40 years in the study by Hashemi et al.).

The results of our also study indicate that mean angle *λ* was significantly smallest in myopia, followed by emmetropia and largest in hyperopia. The differences between the three refraction groups were statistically significant. This is in concord with the findings of Reinstein et al., [26] who measured angle *κ* using Orbscan II at 5.28° ± 1.49° in myopia, 5.77° ± 1.29° in emmetropia and 6.14° ± 1.44° in hyperopia. However, Hashemi et al., [2] also used Orbscan II device but recorded mean angle *κ* in myopia, emmetropia, and hypermetropia at 5.13° ± 1.50°, 5.72° ± 1.10°, and 5.52° ± 1.19°, respectively.

Furthermore, our findings reveal that although angle *λ* was smallest below 20 years of age and largest above 40 years, the differences between age groups was statistically insignificant. Contrarily, London and Wick [3] had reported a substantial decrease in angle with age, but their work focused on changes from birth (angle *λ* measured at 7.88°) to adulthood (angle *λ* measured at 5.08°). Other studies [2, 25] using Orbscan II device have described a significant decrease in angle *κ* with age.

On the other hand, the results of this study show a poor statistical correlation between angle *λ* and all parameters except for a medium negative correlation between angle *λ* and ACD. Therefore, by referring back to our trigonometrical equation [angle *λ* = tan^−1^ AB/BC], it is evident that variation in angle *λ* is more reliant on variation in line BC (ACD + CCT) than on line AB (pupil center offset). In addition, univariate regression analysis demonstrated a significant impact of age, SE, subjective sphere, average *K*, ACD and AL on angle lambda. Multivariate regression showed that the most important determinants of angle *λ* were SE, subjective sphere, ACD, PR and AL. Comparatively, Hashemi et al. [2] found a significant correlation between the refractive status and angle *κ*, which was larger in hyperopes compared to the myopes. Another study by Basmak et al. [7] obtained angle *κ* measurements using both Orbscan II and a synaptophore and reported a significant correlation between the degree of hyperopia and large positive angle *κ* values. Schaeffel [14] however claims the contrary, that there is no correlation between angle *κ* and refraction.

Furthermore, the pupil center offset measurements obtained by the Sirius device in our study reveal that horizontal offset (*x*-axis) was greater in hyperopia than in myopia, whereas the vertical offset (*y*-axis) in both refraction groups was similar. This is confirmed by several other studies [26–30], which all attest to a larger magnitude of horizontal pupil center offset in hyperopes.

We are aware that this work has several study limitations. Firstly, an external A-scan measurement device was required to obtain the ocular axial length needed to calculate angle *λ*. Secondly, our technique needed additional calculation to reach the value of angle *λ*. Over 90% of our participants were myopes, which could have rendered the calculated mean angle *λ* smaller. Moreover, our population sample was relatively young, but we considered this more practical clinically since younger individuals are more likely to seek laser corneal refractive surgery. Furthermore, our study was conducted in a single center and therefore our population sample was limited to a confined geographical area. In addition, our method was not compared to other devices, such as the Orbscan II or the synoptophore, plus the fact that the Sirius device is not the most popular device used in clinical practice.

To conclude, it is strongly recommended that refractive surgeons consider angle *λ* measurements preoperatively, especially when treating hyperopia, to avoid complications of decentered ablation. The Sirius device is a useful tool which combines a Scheimpflug camera to a Placido disc topographer and could serve this purpose well but requires A-scan readings from an external device. Clinically, spectacle prescriptions use the pupil center, which can produce lens decentration and a prismatic effect in the presence of a large angle *λ*. Fortunately, compensatory mechanisms such as convergence and divergence can render this prismatic effect insignificant. On the other hand, in photoablation refractive surgery, even minor decentrations could produce substantial optical aberrations.

## References

[1] Schapero M, Cline D, Hofstetter HW (1968). Dictionary of visual science.

[2] Hashemi H, Khabazkhoob M, Yazdani K (2010). Distribution of angle kappa measurements with Orbscan II in a population-based survey. J Refract Surg.

[3] London R, Wick BC (1982). Changes in angle lambda during growth: theory and clinical applications. Am J Optom Physiol Opt.

[4] Le Grand Y, El Hage SG (1980) Physiological optics. Springer series in optical sciences. p. 73

[5] Irsch K (2015). Optical issues in measuring strabismus. Middle East Afr J Ophthalmol.

[6] Donders FC (1864). On the anomalies of accommodation and refraction of the eye. Translated by Moore WD. Sydenham Society, London

[7] Basmak H, Sahin A, Yildirim N (2007). Measurement of angle kappa with synoptophore and Orbscan II in a normal population. J Refract Surg.

[8] Uozato H, Guyton DL (1987). Centering corneal surgical procedures. Am J Ophthalmol.

[9] Prakash G, Prakash DR, Agarwal A (2011). Predictive factor and kappa angle analysis for visual satisfactions in patients with multifocal IOL implantation. Eye (Lond).

[10] Merrill KS, Lavoie JD, King RA (2004). Positive angle kappa in albinism. J AAPOS.

[11] Scott WE, Mash AJ (1973). Kappa angle measures of strabismic and nonstrabismic individuals. Arch Ophthalmol.

[12] Salmon TO, Thibos LN (2002). Videokeratoscope-line-of-sight misalignment and its effect on measurements of corneal and internal ocular aberrations. J Opt Soc Am A Opt Image Sci Vis.

[13] Von Noorden GK (1996) Binocular vision and ocular motility: theory and management of strabismus. Series C. Mosby, pp 163–167

[14] Schaeffel F (2002). Kappa and Hirschberg ratio measured with an automated video gaze tracker. Optom Vis Sci.

[15] Asano-Kato N, Toda I, Sakai C (2005). Pupil decentration and iris tilting detected by Orbscan: anatomic variations among healthy subjects and influence on outcomes of laser refractive surgeries. J Cataract Refract Surg.

[16] Mandell RB (1994). Apparent pupil displacement in videokeratography. CLAO J.

[17] Savini G, Barboni P, Carbonelli M (2011). Repeatability of automatic measurements by a new Scheimpflug camera combined with Placido topography. J Cataract Refract Surg.

[18] Feng Y, Varikooty J, Simpson TL (2001). Diurnal variation of corneal and corneal epithelial thickness measured using optical coherence tomography. Cornea.

[19] Yeo JH, Moon NJ, Lee JK (2017). Measurement of angle kappa using ultrasound biomicroscopy and corneal topography. Korean J Ophthalmol.

[20] Wachler BS, Korn TS, Chandra NS (2003). Decentration of the optical zone: centering of the pupil versus the coaxially sighted corneal light reflex in LASIK for hyperopia. J Refract Surg.

[21] Pande M, Hillman JS (1993). Optical zone centration in keratorefractive surgery. Entrance pupil center, visual axis, coaxially sighted corneal reflex, or geometric corneal center?. Ophthalmology.

[22] Nepomuceno RL, Boxer Wachler BS, Kim JM (2004). Laser in situ keratomileusis for hyperopia with the LADARVision 4000 with centration on the coaxially sighted corneal light reflex. J Cataract Refract Surg.

[23] York MA, Mandell RB (1969). A new calibration system for photokeratoscopy. II. Corneal contour measurements. Am J Optom Arch Am Acad Optom.

[24] Larsen JS (1971). The sagittal growth of the eye. 1. Ultrasonic measurement of the depth of the anterior chamber from birth to puberty. Acta Ophthalmol (Copenh)..

[25] Gharaee H, Shafiee M, Hoseini R (2014). Angle kappa measurements: normal values in healthy Iranian population obtained with the orbscan II. Iran Red Crescent Med J.

[26] Reinstein DZ, Archer TJ, Rowe EL (2021). Distribution of pupil offset and angle kappa in a refractive surgery preoperative population of 750 myopic, emmetropic, and hyperopic eyes. J Refract Surg.

[27] Erdem U, Muftuoglu O, Gundogan FC (2008). Pupil center shift relative to the coaxially sighted corneal light reflex under natural and pharmacologically dilated conditions. J Refract Surg.

[28] Mabed IS, Saad A, Guilbert E (2014). Measurement of pupil center shift in refractive surgery candidates with Caucasian eyes using infrared pupillometry. J Refract Surg.

[29] Camellin M, Gambino F, Casaro S (2005). Measurement of the spatial shift of the pupil center. J Cataract Refract Surg.

[30] Yang Y, Thompson K, Burns SA (2002). Pupil location under mesopic, photopic, and pharmacologically dilated conditions. Invest Ophthalmol Vis Sci.

